# Persistent Ureteric Dilatation due to Pelvic Actinomycosis Presenting as Pelvic Inflammatory Disease

**DOI:** 10.1155/2011/186708

**Published:** 2011-07-10

**Authors:** Richard Khafagy, Omar Jundi, Karol Rogawski, Siva Namasiviyam

**Affiliations:** Department of Urology, Calderdale Royal Hospital, West Yorkshire, Halifax HX3 0PW, UK

## Abstract

*Actinomyces* is a Gram-positive, filamentous bacterium that normally colonizes mucosal areas. Pelvic actinomycosis is a chronic granulomatous disease caused by *Actinomyces israelii* that frequently mimics ovarian tumors during presentation. It is diagnosed after surgery in most of the cases. Intravenous penicillin is the most preferred therapeutic agent, and it requires hospitalization up to one month. Pelvic actinomycosis is a rare cause of ureteric obstruction and renal failure. The final diagnosis is usually difficult and often apparent only after histological examination of an operative specimen. The present case led us to consider the etiology and clinical findings and to review the management of reported cases involving ureteric obstruction.

## 1. Presentation

The patient to be discussed is a 33-year-old woman who had an intrauterine contraceptive device (IUCD) inserted in 1997. She subsequently presented in December 2009 with pelvic pain and vaginal discharge suggestive of pelvic inflammatory disease. On questioning, she also reported weight loss over the last few months, but no other constitutional symptoms. Clinical examination revealed a fixed pelvic mass, which was initially thought to be malignant. Tumour markers (CA125 and CA153) were normal, and cervical smears in 2008 had been unremarkable. Full blood count was normal, while C-reactive protein was mildly raised. Biochemistry showed a degree of renal insufficiency and reduced eGFR. High vaginal swabs showed a positive growth of *Actinomyces israelii*. Subsequent CT scanning showed bilateral dilatation of the renal pelvis with some tortuosity of the distal ureters on both sides. Bilateral antegrade ureteric stents were inserted through percutaneous nephrostomy tubes to relieve the ureteric obstruction. Retrograde ureteropyelography seven months later again showed a persistently dilated renal pelvis in both kidneys resulting in further stent change. There was loss of calyceal outline with clubbing and ureterohydronephrosis together with a persistently elevated serum creatinine and reduced eGFR that did not normalise to baseline.

## 2. Discussion

Actinomycosis is a rare chronic granulomatous response to infection by the anaerobic Gram-positive bacteria *Actinomyces israelii*. This grows in cellular colonies as filaments which break off, releasing individual organisms. Infection causes a profuse, pus-forming reaction, which drains via multiple small sinuses from the primary abscess. The chronic inflammatory response is able to cross tissue planes, resulting in an appearance often confused with pelvic malignancy. Presentation is often nonspecific, with symptoms such as mild abdominal discomfort, weight loss, and low-grade fevers. Diagnosis is commonly established by selective Fastidious Anaerobic Agar culture and the presence of microscopic sulphur granules. However, often the diagnosis is not considered until the postoperative stage. Risk factors include a history of abdominal surgery, ingestion of foreign bodies, or the presence of an IUCD. 

Treatment involves a six-month course of penicillin antibiotic therapy. A common complication associated with pelvic actinomycosis is ureteric stricture, which may resolve upon completion of antimicrobial therapy [1]. Several cases have involved intra-abdominal abscess or ischiorectal abscess that required ureteric stenting to relieve the ureteric obstruction [2]. Use of immunosuppressants has also been described to mediate the chronic inflammatory process, thereby limiting diffuse peritoneal disease [3].

Pelvic actinomycosis is often related to the presence of a foreign body. It has been theorised that the presence of such a body and the anaerobic flora of the vagina provide a perfect setting for actinomycotic growth, which is possible with all types of IUCDs. In this case, laparotomy was avoided when cultures grew *actinomyces*, but often the diagnosis is not made until the postoperative stage. Once diagnosed, antibiotic therapy, at an initially high dosage, is recommended.

This paper highlights the chronic nature of Actinomycosis involving the ureters. The patient still had evidence of ureteric dilatation up to seven months after diagnosis. Functional studies were not performed, so the obstruction was not demonstrated, but the persistently reduced eGFR suggests that there was a permanent loss of functional renal tissue. We suggest that the resolution of the ureteric dilatation may not be immediate and that an alternative method of long-term drainage for the kidneys should be sought.
